# Case of Aortic Dissection Diagnosed by Suprasternal Notch Point-of-Care Ultrasound (POCUS) in a 73-Year-Old Man With Chest and Lower Extremity Pain

**DOI:** 10.7759/cureus.110529

**Published:** 2026-06-09

**Authors:** Simon Londono, Franz C Mendoza-Garcia, Brian Kohen

**Affiliations:** 1 Emergency Medicine, Memorial Healthcare, Pembroke Pines, USA

**Keywords:** aortic dissection, pocus, suprasternal notch view, suprasternal ultrasonography, type a aortic dissection

## Abstract

Early detection of aortic dissection is critical, as it can be quite elusive. Suprasternal notch (SSN) point-of-care ultrasound (POCUS) is underutilized in emergency department settings. A 73-year-old man with hypertension, hyperlipidemia, smoking history, and a known aortic aneurysm presented with two hours of severe left-sided chest pain, left leg pain, numbness, and weakness. Physical examination revealed an irregular, slow pulse and a non-palpable left posterior tibial pulse. The electrocardiogram showed atrial fibrillation with slow ventricular response. POCUS parasternal view revealed a dilated aortic root; SSN view, performed by an intern, clearly demonstrated an intimal flap. Computed tomography angiography (CTA) confirmed Stanford Type A dissection extending to the renal arteries and occluding the left common iliac artery. Emergent cardiothoracic and vascular surgery consultation and transfer for definitive operative management were initiated before CTA results.

This case highlights the diagnostic utility of SSN POCUS, especially in novice hands, for rapid identification of life-threatening aortic pathology.

## Introduction

Aortic dissection is a vascular emergency associated with significant morbidity and mortality, with outcomes closely tied to the speed of diagnosis and intervention. According to the Stanford classification system, Type A dissections involve the ascending aorta, while Type B does not. Mortality increases by approximately 1%-2% per hour following symptom onset in untreated Stanford Type A dissections [1]. Computed tomography angiography (CTA) remains the diagnostic gold standard; however, delays related to diagnostic uncertainty, imaging availability, or patient instability may adversely affect outcomes [2].

Point-of-care ultrasound (POCUS) has emerged as a valuable adjunct in the early evaluation of suspected aortic pathology. Common sonographic findings include aortic root dilation, pericardial effusion, and direct visualization of an intimal flap [3]. However, standard transthoracic echocardiographic windows may inadequately visualize the aortic arch and descending thoracic aorta [4].

The suprasternal notch (SSN) view permits longitudinal imaging of the aortic arch and its major branch vessels, offering diagnostic information not available through routine parasternal or apical views. This view is obtained by placing the phased array ultrasound probe above the sternal notch and fanning caudally until the aortic arch is seen. Although image acquisition may be limited by factors such as restricted neck mobility, patient discomfort, body habitus, or emergency department bed equipment, prior studies have demonstrated high feasibility, with emergency physicians successfully obtaining SSN views in up to 97% of patients undergoing POCUS evaluation [5]. Previous case reports have described the diagnosis of aortic dissection using SSN POCUS in the emergency department setting [6-8]; however, this sonographic window remains underutilized and inconsistently incorporated into ultrasound training, particularly among novice operators. We present a case of Stanford Type A aortic dissection initially identified using SSN view POCUS performed by an emergency medicine intern, in a patient with an atypical presentation.

## Case presentation

A 73-year-old man with a history of hypertension, hyperlipidemia, cigarette smoking, and a known thoracic aortic aneurysm presented to the emergency department with sudden-onset severe left-sided chest pain radiating to the left lower extremity after awakening from sleep. His symptoms were accompanied by left leg numbness and weakness.

On examination, the patient appeared in significant distress. Vital signs revealed an irregular bradycardic pulse, and cardiac monitoring demonstrated atrial fibrillation with a slow ventricular response. Peripheral vascular examination revealed an absent left posterior tibial pulse. No focal neurologic deficits were identified aside from subjective sensory changes in the left leg.

POCUS was performed early in the patient’s evaluation. A parasternal long-axis view demonstrated a dilated aortic root with a linear echogenic structure concerning for an intimal flap (Figure 1; Video 1). An SSN view, obtained by an emergency medicine intern under supervision, clearly demonstrated a mobile intimal flap within the aortic arch (Figure 2; Videos 2-3).

**Figure 1:**
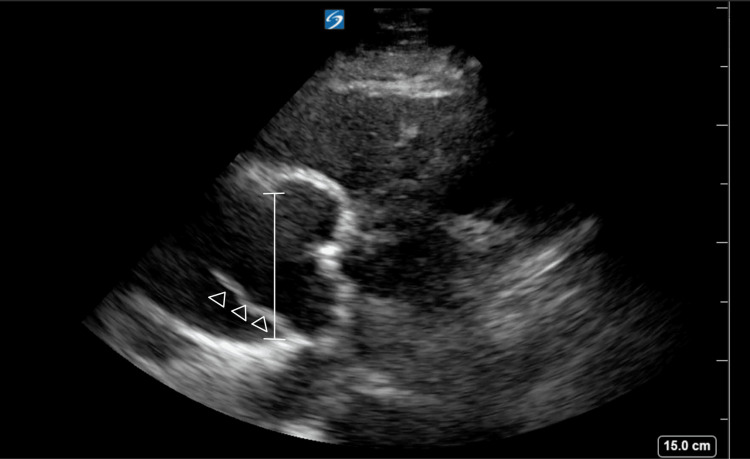
Enlarged aortic root at the sinotubular junction with a dissection flap extending into the aortic valve cusp. White triangles correspond to intimal flap dissection of the aorta.

**Video 1:** Parasternal long-axis (PSLA) view demonstrating an aortic intimal flap with spontaneous echo contrast visible in the right ventricle.

**Figure 2:**
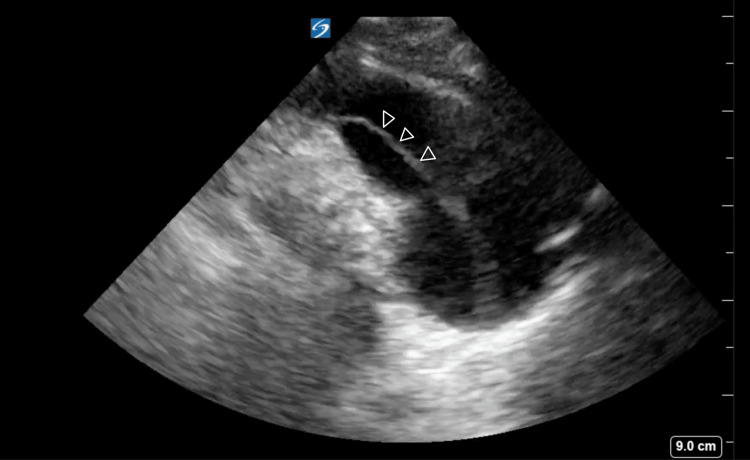
Descending aorta demonstrating an aortic dissection flap. White triangles correspond to intimal flap dissection of the aorta.

**Video 2:** Suprasternal notch view (SSNV) demonstrating an aortic dissection within the aortic arch, with the right brachiocephalic artery visualized branching from the aorta.

**Video 3:** Suprasternal notch view (SSNV) demonstrating an aortic dissection flap within the descending aorta.

CTA of the chest, abdomen, and pelvis confirmed a Stanford Type A aortic dissection extending distally to the renal arteries, with occlusion of the left common iliac artery (Figures 3-6). Cardiothoracic and vascular surgery were consulted prior to the availability of CTA results. The patient was emergently transferred for operative management, where he underwent cardiopulmonary bypass, repair of the aortic dissection, and aortic valve replacement. His postoperative course was notable for resolution of the limb ischemia and acute kidney injury. He was discharged home after a nine-day hospitalization and subsequently completed rehabilitation.

**Figure 3:**
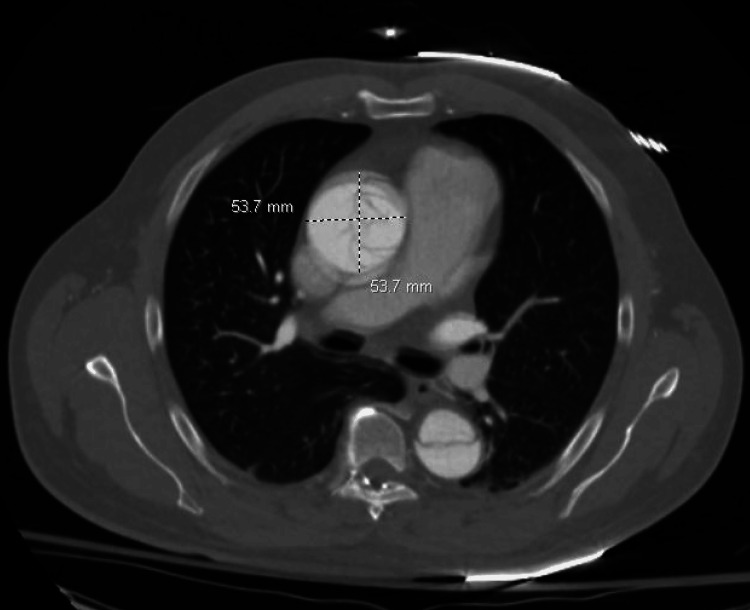
Transverse CT view of the ascending aorta demonstrating aortic dissection. CT, computed tomography

**Figure 4:**
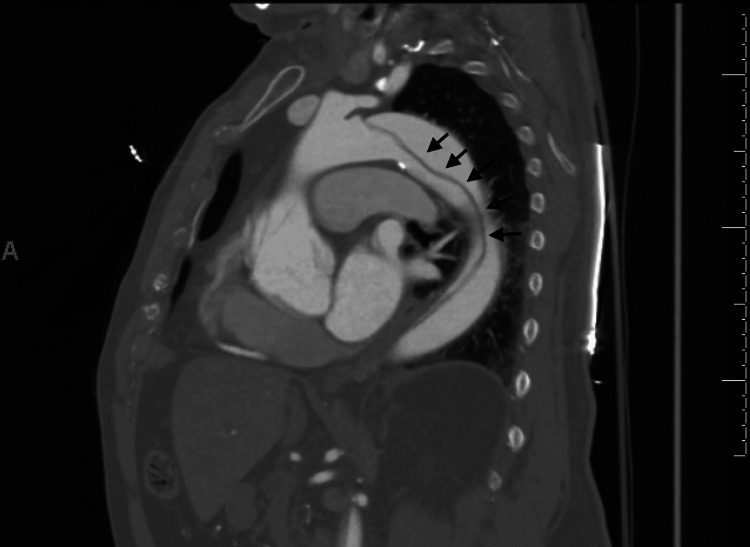
Sagittal CT view demonstrating aortic dissection. Black arrows correspond to intimal flap dissection of the aorta. CT, computed tomography

**Figure 5:**
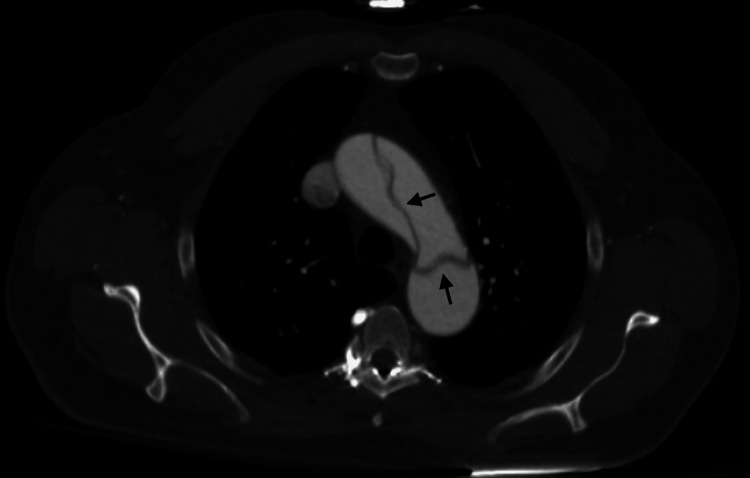
Transverse CT view of the aortic arch demonstrating a dissection flap. Black arrows correspond to intimal flap dissection of the aorta. CT, computed tomography

**Figure 6:**
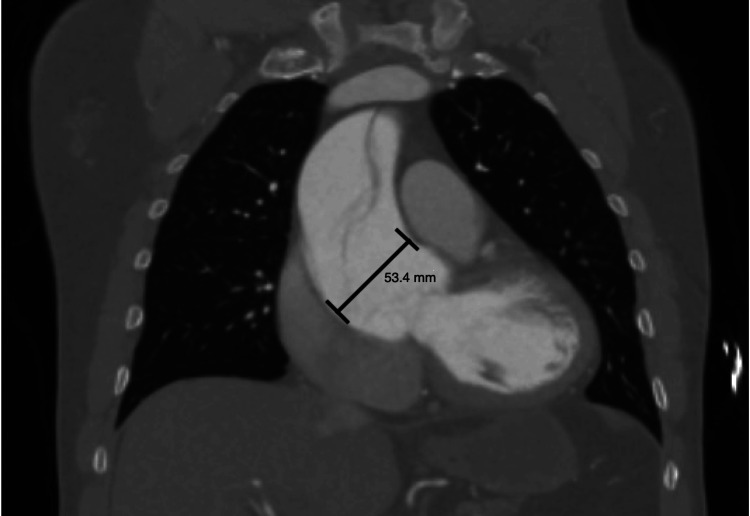
CT measurement of the dilated ascending aorta. CT, computed tomography

## Discussion

This case highlights the value of SSN POCUS as an early diagnostic adjunct in suspected aortic dissection and demonstrates its potential impact on clinical decision-making in the emergency department. Aortic dissection should be suspected in any patient presenting with chest pain and a known history of a thoracic aneurysm. In this instance, SSN POCUS was not merely confirmatory but directly influenced diagnostic prioritization and expedited definitive management.

The patient’s presentation was diagnostically challenging. Although chest pain with limb ischemia could suggest acute vascular pathology, the presence of atrial fibrillation with a slow ventricular response introduced diagnostic ambiguity and could have redirected evaluation toward primary arrhythmia or cardioembolic disease. While atrial fibrillation has been described in association with aortic dissection, bradycardic presentations are uncommon and may delay recognition of this life-threatening condition [7].

Initial parasternal POCUS raised suspicion for aortic pathology by demonstrating a dilated aortic root, but only after incorporating the SSN view was the diagnosis clinched. This underutilized view directly visualized the mobile intimal flap in the aortic arch.

A literature review performed through February 2026 shows that there have only been two published case reports describing a Type A aortic dissection diagnosed by SSN POCUS by the emergency medicine physician [6,8]. The present case adds to this scarce literature by illustrating that SSN imaging can be successfully performed by an intern-level resident with minimal formal ultrasound training and can remain diagnostically valuable even in the setting of an atypical presentation.

## Conclusions

SSN POCUS is an underutilized but powerful adjunct for the early detection of aortic dissection. Emergency physicians should consider adding this view to their cardiac POCUS in patients presenting with chest pain, pulse deficits, or unexplained hemodynamic abnormalities. Training novice sonographers in this technique may lead to earlier diagnosis and improved clinical outcomes.
